# The Effect of Photoperiod Genes and Flowering Time on Yield and Yield Stability in Durum Wheat

**DOI:** 10.3390/plants9121723

**Published:** 2020-12-07

**Authors:** Jose M. Arjona, Dolors Villegas, Karim Ammar, Susanne Dreisigacker, Christian Alfaro, Conxita Royo

**Affiliations:** 1 IRTA (Institute for Food and Agricultural Research and Technology), Sustainable Field Crops Program, 25198 Lleida, Spain; chemarjona@gmail.com (J.M.A.); conxita.royo@irta.cat (C.R.); 2 International Maize and Wheat Improvement Centre (CIMMYT), Mexico City 06600, Mexico; k.ammar@CGIAR.ORG (K.A.); S.Dreisigacker@CGIAR.ORG (S.D.); 3 Instituto de Investigaciones Agropecuarias, Centro Regional de Investigación INIA-Rayentue, Rengo 2940000, Chile; calfaro@inia.cl

**Keywords:** earliness per se, day length, solar radiation, grain filling, grain weight, grain number

## Abstract

This study analysed the effect of flowering time as influenced by photoperiod sensitivity genes on yield and yield stability in durum wheat. Twenty-three spring genotypes harbouring different allele combinations at *Ppd-A1* and *Ppd-B1* were grown in 15 field experiments at three sites at latitudes from 41° to 19° N (Spain, Mexico-North and Mexico-South). Low temperature and solar radiation before flowering and long day length during grain-filling characteristic for the Spanish site resulted in high grain number/m^2^ (GN) and yield (GY), while a moderate GN combined with high solar radiation during grain-filling at Mexico-North led to heavier grains. Allele combination GS100-*Ppd-A1a*/*Ppd-B1a* reduced the flowering time up to nine days when compared with *Ppd-A1b*/*Ppd-B1a*. Differences in flowering time caused by *Ppd-A1*/*Ppd-B1* allele combinations did not affect yield. Combinations GS105-*Ppd-A1a*/*Ppd-B1b* and *Ppd-A1b*/*Ppd-B1b* resulted in the highest GN, linked to spikelets/spike, which was higher in GS105-*Ppd-A1a*/*Ppd-B1b* due to more grains/spikelet. Flowering time caused by *Eps* had a minor effect on GN, spikes/m^2^ and grains/spike, but late flowering resulted in reduced grain weight and GY. Allele combinations harbouring alleles conferring a similar photoperiod sensitivity response at *Ppd-A1* and *Ppd-B1* resulted in greater yield stability than combinations that carry alleles conferring a different response. Allele combination GS100-*Ppd-A1a*/*Ppd-B1a* was the most suitable in terms of yield and yield stability of durum wheat cultivated under irrigation within the studied latitudes.

## 1. Introduction

Wheat is a basic food providing near 20% of the calories of the human population [1]. Durum wheat (*Triticum turgidum* L. ssp. *durum*) stands for about 10% of global production of wheat. In Mediterranean environments durum wheat is generally cultivated under rain-fed conditions, in which yield is strongly affected by unpredictable fluctuations of temperature and erratic rainfall patterns across growing seasons. In a context of climate change, wheat yields are expected to fall as a consequence of rising temperatures, and more extreme weather events are predicted for the next few decades [2]. A decrease in global wheat production of between 4.1% and 6.4% is expected for each °C of temperature increase [3]. In this framework, understanding the genetic and environmental factors affecting yield formation is paramount for targeting breeding strategies to release new cultivars adapted to the upcoming environmental scenarios, thus ensuring food security [4].

Grain yield is a very intricate trait governed by environment, genotype, and the interaction between the two. Given the considerable environmental effect on final yield, great variability between sites and crop seasons is generally important The importance of yield stability in durum wheat has been highlighted in previous studies [5,6], and there is a general consensus among breeders to pursue high-yielding and stable varieties. Whereas the concept of high yield is clear, there are several definitions and calculation methods for yield stability [7]. Stability involves two different concepts: static (or biological) stability and dynamic (or agronomic) stability. A genotype is considered statically stable when its performance remains unaffected notwithstanding the environmental variation, so the mean value of a phenotypic trait with static stability would not change between different conditions [8]. The static concept is the most valuable for some quality or disease-resistance traits when the same response is desirable in all target environments. On the other hand, for yield performance, dynamic stability is generally sought. Agronomic stability is defined as the superior performance of a given genotype in different environments, as mean values for each environment being different. In the current study we use the concept of dynamic stability, while adaptability is considered to be the ability to perform well in all testing environments. The incorporation of dynamic stability in the new varieties released by breeding programs would increase the resilience to the environmental variations derived from climate change.

Several parametric and non-parametric statistical methods are available for analysing yield stability. Parametric methods observe the genotypic responses of a sample to environmental conditions. Among the most common of these are the regression slope (*b_i_*), which provides information on both stability and yield performance [9], the Lin and Binns [10] superiority measure (*Pi*), which prioritises high-yielding genotypes and shows high values for those performing well in most environments, the Shukla’s [11] stability variance (*σ*^2^) and the Wricke’s [12] ecovalence (*W_i_*^2^) that emphasise yield stability against yield performance, with the closest values to zero denoting great stability. Non-parametric methods—including Kang’s [13] yield stability (*YS*)—describe genotypic performance over ranks of data relative to environmental factors. In breeding programs, selection for stability-low variance does not necessarily results in high yield, and an increased yield may, therefore, not be linked to stability when these parameters are used. Therefore selection based only in one of these parameters has shown an increased type II error, which would allow selecting genotypes that are neither stable nor high-yielding [13]. For this reason Kang [14] proposed a non-parametric method of rank-sum, which later developed in more detail, where both yield and the stability-variance are taken into account. It was named yield stability (YS) [13] or yield reliability [15].

Grain yield in wheat can be considered as the multiplication of two main yield components, grain weight (GW) and grain number per unit area (GN), which appear sequentially during crop development. Variation in yield components are closely related to environmental conditions, especially those before and around flowering time, the most sensitive stage to environmental variations. Flowering time is a crucial stage delimiting the period of spike formation and the onset of the grain-filling period, when the number of grains/spike and GW are defined. Time to flowering is considered a major trait defining wheat adaptation to a particular array of growing conditions [16,17] and a critical feature for increasing resilience to weather variations and ultimately achieving high yields [18]. Wheat phenology must be fine-tuned to a given environment to find the appropriate earliness to avoid excessively high temperatures during flowering time that would affect grain number by reducing fertility [19], but without risking frost damage during post-heading phases [20]. It has been suggested that changes in allele frequencies in loci responsible for day length and temperature responses will be critical for the adaptation of cropping systems to climate change [21].

The complex genetic control of phenology is mainly based on photoperiod sensitivity genes (*Ppd-1*), vernalisation requirement genes (*Vrn-1*), and earliness per se (*Eps*) [22]. The vernalisation requirement is regulated by the *Vrn-1* genes. In durum wheat these comprise homologous copies designated as *Vrn-A1* and *Vrn-B1* and positioned in chromosomes 5A and 5B, respectively [23,24]. In wheat, photoperiod sensitivity is regulated by *Ppd‑1* genes, which in durum wheat are *Ppd-A1* and *Ppd-B1*. The *Ppd-A1* gene has three alleles described: *Ppd-A1b* considered the wild type linked to photoperiod sensitivity and two alleles (*GS100* and *GS105*) causing photoperiod insensitivity [25]. The *Ppd-B1* gene has two alleles, the wild type producing photoperiod sensitivity (*Ppd-B1b*) and *Ppd-B1a* causing photoperiod insensitivity [26,27]. Alleles at *Ppd-A1* causing photoperiod insensitivity have a stronger effect on shortening the pre-flowering period than the *Ppd-B1* alleles (*GS100* > *GS105* > *Ppd‑B1a*) [27]. The effect of *Eps* on development rate is independent of vernalisation and photoperiod [17,22]. Less is known about the genes that underpin *Eps* compared to *Vrn-1* and *Ppd-1*, and the majority of what is known is derived from studies of diploid wheats such as *T. monococcum* L.

A mega-environment is, according to Braun et al. [28] a ‘broad, not necessarily continuous often transcontinental area with similar biotic and abiotic stresses, cropping systems and consumer preferences’. Vernalisation alleles, and to a lesser extent photoperiod sensitivity genes, are key classification criteria for for adaptability to a given mega-environments. Photoperiod sensitivity may be considered a mechanism for fine-tuning the optimal flowering time in a given environment within some mega-environments. In durum wheat, most varieties have the *Vrn-A1c* allele [29], corresponding to the spring growth habit, called the ‘Langdon type’, with rather rare alleles existing in genotypes from Russia, Ukraine, Azerbaijan and Hungary [30]. Therefore, once the appropriate *Vrn* alleles have been defined, a good strategy for adjusting flowering time to a given target environment would be to use a suitable combination of photoperiod sensitivity alleles. Allele combinations at *Ppd-1* genes may produce variations in flowering time of up to 40 days in bread wheat [31] and up to 20 days in durum wheat in certain environments [27]. A recent study has shown interaction between *Ppd-1* genes in durum wheat substantially modify flowering date depending on the allele combination [32]. In addition to the actual allelic composition at the *Ppd-1* loci, copy number at *Ppd-B1* has been shown to influence on heading date in durum wheat [33]. In bread wheat the interaction between *Ppd-D1* and *Ppd-B1* has been demonstrated by Tanio and Kato [31] and Bentley et al. [34], among others. Yield advantages may result from photoperiod insensitivity which have been assessed as 15% in Central Europe and over 35% in Southern Europe [35].

The present study is part of a comprehensive project, aiming to understand the effect of flowering time, as regulated by photoperiod sensitivity, on the adaptability and productivity of durum wheat. For this purpose, a collection of durum wheat genotypes involving known allele combinations for major genes at the *Ppd-A1* and *Ppd-B1* loci and with great variation in flowering time also due to earliness per se was developed and tested under contrasting northern latitudes (all below 45° N). Results are presented on the effect of allele combinations at *Ppd-A1* and *Ppd-B1* on detailed yield components, yield stability, and the variability induced by genetic factors other than *Ppd-1* genes (putatively *Eps*). Previous results allowed quantifying the limitations imposed by day length, temperature and solar radiation on yield [36], and to assess the effect of *Ppd-1* genes on the pattern of development [27], biomass production and allocation [37] and grain filling [38]. Arjona et al. [39] analyzed the effect of the alleles independently on yield and yield components, while the current study goes a step further analyzing the combination of allele variants at the two genes. The study of Royo et al. [37] analyzed the effect of allele combinations on productivity with a physiological approach considering yield as the product of biomass and harvest index. However, the allele combination effect on the approach expressing yield as the product of its components (i.e., spikes/m^2^, grains/spike, grain weight) and sub-components (grains/spike divided into spikelets/spike and grains/spikelet) is still unexplored. Some adaptability aspects remain unknown, such as the interaction between site and allele combination, and yield stability as affected by *Ppd-1* allele combinations, or the effects of *Eps* on yield and yield components.

This study was conceived under the hypothesis that the alterations of flowering time caused by *Ppd-A1*/*Ppd-B1* allele combinations and *Eps* result in exposing wheat to different environmental conditions during its development, thus affecting yield formation and possibly yield stability. In order to test this hypothesis the current study aimed to: (i) analyse the effect of changes in flowering time associated to *Ppd-A1*/*Ppd-B1* allele combinations on yield and yield stability at three sites of contrasting northern latitude, (ii) identify the most suitable allele combination at each site in terms of yield and yield stability, and (iii) explore the relationships between flowering time due to putative *Eps* and yield formation.

## 2. Results

### 2.1. Environmental and Genetic Effects

**Figure 1:**
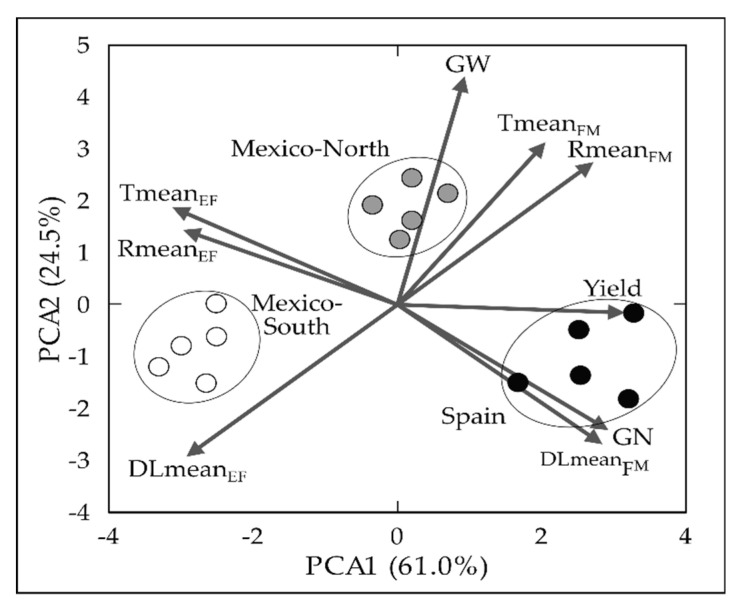
Biplot of the first two axes of the principal component analysis summarizing the relationships between environmental variables and yield components for the three experimental sites. Eigenvalues of the correlation matrix are represented as vectors symbolizing environmental variables and yield associated traits. DLmean_EF_: mean day length from emergence to flowering, DLmean_FM_: mean day length from flowering to maturity, Tmean_EF_: average of mean daily temperatures from emergence to flowering, Tmean_FM_: average of mean daily temperatures from flowering to maturity, Rmean_EF_, mean of daily solar radiation from emergence to flowering, Rmean_FM_ mean of daily solar radiation from flowering to maturity, GN: grain number/m^2^, GW: grain weight, GY: grain yield. Points correspond to average genotype values for each year at each site. Black dots: Spain; Grey dots: Mexico-North; White dots: Mexico-South.

Principal component analysis (PCA) was conducted with the environmental variables and yield-related traits calculated for each plot. Environmental variables and yield components were clearly different at each site. The location of the points corresponding to the experiments conducted in Spain indicates that this site was characterised by low temperature and radiation before flowering, long photoperiod during grain filling and a high GN and GY. The Mexico-South site showed a high photoperiod, temperature and radiation until flowering, and the lowest GY, while Mexico-North, which showed the highest temperature and radiation during grain filling, had the shortest day length from emergence to flowering, resulted in the production of the heaviest grains. In all cases, yearly variations were much lower than differences between sites, as shown by the clustering in the PCA biplot of the points corresponding to the five years at each site (Figure 1).

Table 1 shows a summary of the allele combinations for *Ppd-A1* and *Ppd-B1* loci studied in this work.

The genotype effect was statistically significant for all traits and accounted for 3.1% of the total variation for days from emergence to flowering, the trait with the smallest genotypic control and for up to 55.8% of total variation for GW, the trait under strongest genotypic control. The partitioning of the genotype effect into its components, revealed that the allele combination at *Ppd-1* only affected flowering time and GN but had no effect on grain filling duration, GW or GY. The genotype × site interaction was significant for all traits except GN (Table 2).

**Figure 2:**
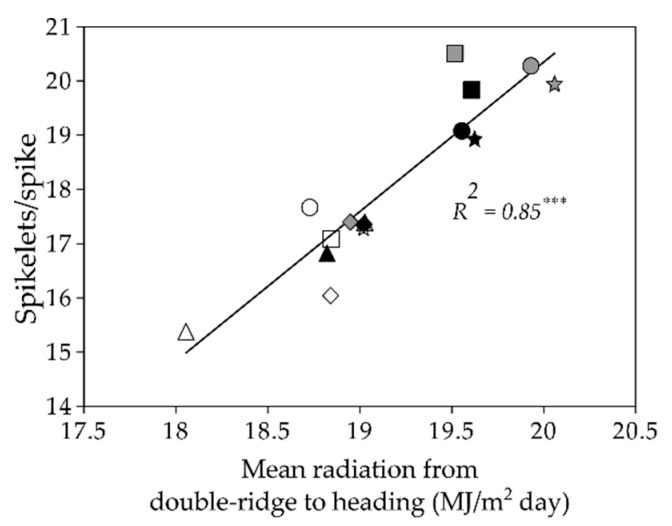
Relationship between mean daily solar radiation from double-ridge to heading and the number of spikelets/spike. Each point represents the mean of 3-9 durum wheat genotypes averaged over 3 years at each site. Allele combinations at *Ppd-A1* and *Ppd-B1* loci are represented with the following symbols (see Table 1 for allele combination acronyms). △: I0I; ◇: I5I; □: I5S; ☆: SI; ○: SS. Black symbols: Spain; Grey symbols: Mexico-North; White symbols: Mexico-South. *** *P* < 0.001.

There were no statistically significant differences between allele combinations for GY at any site. At all sites, the largest GN and the smallest GW values corresponded to combination I5S, but it did not differ from combination SS for any of these traits. The largest GN for I5S was due to higher grains/spike, although the differences were not statistically significant in Mexico-South (Table 3). Significant differences for the number of spikelets/spike and grains/spikelet were detected between allele combinations across sites. (Table 3). Allele combination I5S reached on average the highest absolute values for both spikelets/spike and grains/spikelet. A significant and positive relationship was found between mean solar radiation from double ridge to heading and spikelets/spike when they were calculated at each site with the mean values of each allele combination across years (Figure 2). Grain setting was analysed in Spain, and no differences were found between allele combinations (Supplementary Table S4). In summary, allele combination affected GN and GW in opposite directions, with significantly different behaviour of combination I5S, but with no significant effect on GY.

### 2.2. Relationships between Phenology Variation Due to Eps and Yield Formation

**Figure 3:**
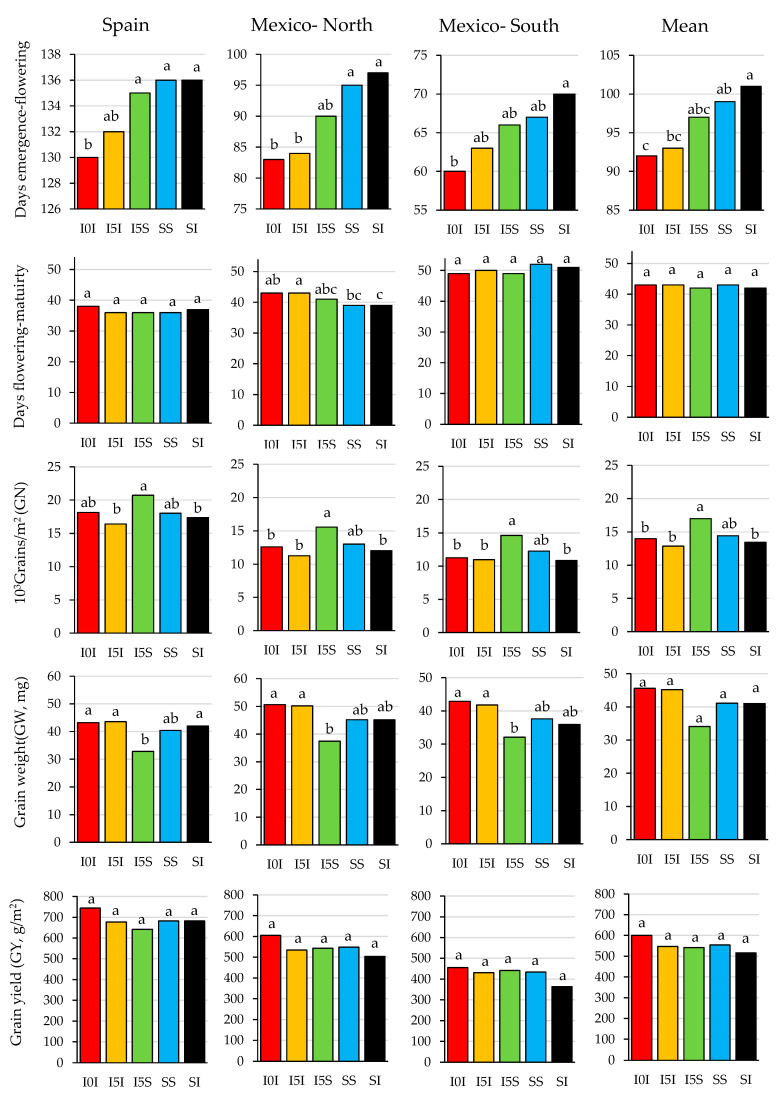
Means comparison for phenology and yield related traits in a set of 23 durum wheat genotypes grouped according to five allele combinations at *Ppd-A1* and *Ppd-B1* loci and tested during five years at three sites of contrasting northern latitude. Means within each graph with different letters are significantly different for a least significant differences (LSD) test at *P* < 0.05. See Table 1 for acronym description.

Although allele combinations at *Ppd-1* had no effect on GY within or across sites (Figure 3), the analysis of variance (ANOVA) showed strong variability for flowering time and GY within them (Table 2). For this reason, the relationships between phenology and yield-related traits were explored, without considering the effect of the allele combination. Thus, Pearson correlation coefficients were calculated with the best linear unbiased predictor (BLUP) values after removing the effect of the allele variants at *Ppd-1* by site and year. The results showed that, in general, the changes in flowering date within allele combinations did not affect GN, and the number of days from flowering to maturity did not affect yield-related traits (Table 4). However, long emergence to flowering periods were negatively associated with GW, and in 12 out of 15 experiments they were also negatively associated with GY (Table 4).

**Figure 4:**
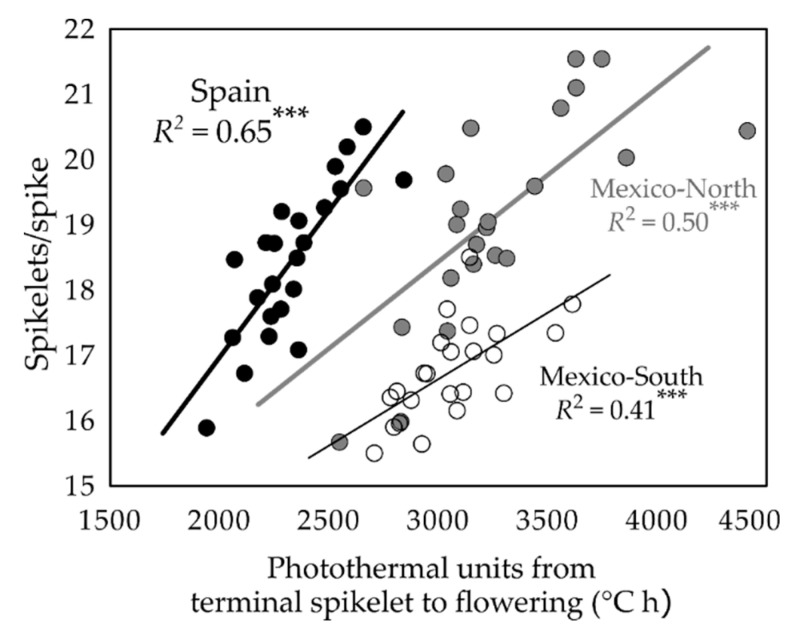
Relationships between photo-thermal units following Dalezios et al. (2002) from terminal spikelet to flowering and the number of spikelets/spike at each experimental site. Each point is the average value of the BLUPs of each of 23 durum wheat genotypes across three years considering flowering time due to *Eps*. Black symbols: Spain; Grey symbols: Mexico-North; White symbols: Mexico-South. *** *P* < 0.001.

The number of days for emergence-flowering did not affect spike number or the number of grains/spike but was positively and significantly associated with the number of spikelets/spike in eight out of nine experiments (Table 5). Grain number depended on the number of grains/spike and on its components, grains/spikelet and spikelets/spike, as they were significantly correlated with GN in seven out of nine experiments (Table 5). The number of spikelets/spike was positively and significantly correlated with photo-thermal units from terminal spikelet to flowering (Figure 4). In summary, spikelets/spike was increased in late flowering genotypes, but GN was not directly dependent on spikelets/spike at all sites.

### 2.3. Ppd-1 Allele Combinations and Yield Stability

The five yield stability indices calculated gave consistent results (Table 6). The slopes of the joint regression analysis (*b*) ranged between 0.87 for allele combination I5S and 1.19 for allele combination SI, the latter value being the only one significantly different from 1. The superiority measure (*Pi*) was significantly different between allele combinations, with the lowest and highest value corresponding to combinations I0I and SI, respectively. This indicates that allele combination I0I was close to the best yielding standard and allele combination SI to the worst. Values for Shukla’s stability, *σ*^2^, that were not significantly different from 0 appeared for allele combinations I0I, I5I and SS, suggesting that they were the most stable. Wricke’s ecovalence revealed similar patterns, recognizing allele combinations I5I and SS as showing the highest stability. Kang’s YS_i_ was in accordance with the previous indices, indicating that the I5S and SI allele combinations had a worse ratio between stability and yield than the pool formed by I0I, I5I and SS.

## 3. Discussion

The wide range of latitudes, but all below 45°, and the different environmental conditions of the three experimental sites used in this study, resulted in the site effect being the most relevant in the ANOVA model for explaining phenotypic variability for all the analysed traits except GW. Although other factors such as soil characteristics and agricultural practices could affect variability between sites, the results of the PCA indicated that 85.5% of the information contained in the environmental data and yield attributes was explained by the two first axes of the multivariate analysis. The biplot of the PCA revealed that the environmental dissimilarities led to contrasting yield formation pathways in the different testing sites. The low temperatures and solar radiation before flowering and the large day length during grain filling were characteristic of the Spanish site and led to the highest GN and GY. This result is in agreement with the interaction between the effect of temperature and solar radiation on GN during the pre heading phase reported elsewhere [40] and the positive effect of low temperatures in pre-anthesis on GN [36]. In contrast, the low day length before flowering and the high solar radiation during grain filling recorded in Mexico-North produced the heaviest grains, while the high temperature, solar radiation and day length before flowering and the low temperature and radiation during grain filling characteristic of the spring planting in Mexico-South led to the lowest yields. The importance of solar radiation during grain filling for achieving high GW has been reported previously [36]. In contrast, the variability induced by the year effect was lower than that caused by the site, as revealed by the ANOVA and PCA.

Although the genotype had a much lower effect than the site for explaining differences in phenology, genotypes differed significantly in their phenological pattern. A certain bias could exist in the analysis of allele combinations, due to the shared background of the lines. However, the “genotype within allele combination” variation was used as an error term in the test of allele combination effects, which was considered a strict enough threshold to cover this possible deviation. The longest period from emergence to flowering was consistently recorded for allele combinations *Ppd-A1b/Ppd-B1a* (SI) and *Ppd-A1b/Ppd-B1b* (SS), as shown previously [41], but without significant differences with allele combination GS105-*Ppd-A1a/Ppd-B1b* (I5S). Across sites and in Spain and Mexico-South individually, the allele combination had no effect on grain filling duration. The interaction allele combination × site was significant according to the ANOVA due to the different duration of grain filling in Mexico-North, where differences between allele combinations were maximised. Quantifying the effect of any given allelic combination is an important prerequisite to determine its value for breeders and ultimately whether it should be selected for or against. The identification of sites, such as the CIMMYT station of Obregon (Mexico-North), where these allele combinations-related differences on grain filing duration are maximised, and how these relate to yield formation, is an important piece of information for establishing future studies assessing allele effects on yield components and formation. This is all the more important given that this particular Mexico-North location has been shown to be highly representative of many irrigated environments around the world, a production system representing some 32 million hectares worldwide [42]. Therefore, it is reasonable to expect that any allelic effect of major phenology genes would have similar effect in Obregon as in the vast majority of the irrigated environments worldwide.

In contrast with the relatively low effect of the genotype on crop phenology, genotypic differences accounted for 55.8%, 27.5% and 11.0% of the total variation observed for GW, GN and GY, respectively. The known strong genetic control of GW [36,39] and GN [43,44,45] is in agreement with these results. The subdivision of the genotype variability into its components revealed that within the range of environments studied, major genes regulating flowering time had no effect on GY at any site or across them. However, differences between the *Ppd-A1*/*Ppd-B1* allele combinations were significant for GN, with combinations I5S (GS105-*PpdA1a/Ppd-B1b*) and SS (*Ppd-A1b*/*Ppd-B1b*) consistently tending to have more grains per unit area than the remaining ones. The detailed analysis conducted to elucidate the reason for the largest GN recorded in genotypes carrying the allele *Ppd-B1b* causing photoperiod sensitivity revealed that it was due to a higher number of grains/spike, as differences were not found for number of spikes per unit area. However, the intensity of the effect of the *Ppd-B1b* allele depended on the allele present at *Ppd-A1*, as the number of grains/spike was consistently higher for combination I5S (GS105-*Ppd-A1a*/*Ppd-B1b*) than for SS (*Ppd-A1b*/*Ppd-B1b*). The dissection of the components of the number of grains/spike revealed that the number of spikelets/spike was similar in both combinations across sites (19.1 and 19.0 for allele combinations I5S and SS, respectively) and at each site, in agreement with the similar mean solar radiation received from double ridge to heading by genotypes carrying one of the two allele combinations. However, although differences in the number of grains/spikelet were not statistically significant at any site, they were significant across them, with I5S having 15% more grains/spikelet than SS (from 14% to 21% depending on the site). The earlier flowering time of I5S (from 1 to 5 days depending on the site) may have provided better environmental conditions for grain setting once the number of spikelets was set [46,47], thus resulting in a higher number of grains/spikelet. A previous study showed that when allele combinations carrying the *Ppd-B1a* allele (I0I, I5I and SI) were compared with those carrying allele *Ppd-B1b* (I5S and SS), the latter caused an increase in the number of grains/spike due to a consistently higher number of spikelets/spike [39]. The current study went a step further by analysing differences between alleles GS105-*Ppd-A1a* and *Ppd-A1b* in the genetic background of *Ppd-B1b*, and the results showed that the number of grains/spikelet was more relevant than the number of spikelets/spike in the increase in number of grains/spike.

A further analysis of the relationship between yield formation and phenology was conducted considering the variability in flowering time existing within allele combinations, i.e., the effect of *Eps*. The large variability for flowering time within any given allele combinations was not surprising, as a previous study revealed the presence of strong *Eps* in the studied genotypes [27].

Correlation analyses showed that flowering time had a lower effect on GN, as correlation coefficients between the two traits were only significant in two of the 15 experiments. In contrast, late flowering had a consistently negative effect on GW, as observed by other authors [40,48], and on GY. Grain filling duration had a minor effect on yield formation. These results indicate that, in the range of environments studied, early flowering was a favourable trait for achieving high yields. The negative effect of late flowering on GW and yield could be due to the higher temperatures suffered during grain filling by late flowering genotypes, as they may have limited the allocation of resources to grains, resulting in lower productivities [49,50,51]. The lack of significant differences between allele combinations for GY, even though the late flowering genotypes were clearly handicapped in their yield potential, may have been due to the aforementioned importance of *Eps*, with each allele class having a very wide range of phenologies, including some earlier types that were not penalised in their yield. This *Eps* would, therefore, be responsible for off-setting the potentially negative effect of major *Ppd* alleles that otherwise promote late flowering and, therefore, less than optimal yield formation. Other authors have confirmed the close relationship between phenology and yield in durum wheat. Milner et al., using a representative durum wheat multiparental cross population detected overlapping QTL for these two traits, and yield QTLs not associated to phenology had minor genetic effects [52].

The dissection of GN into its components revealed that the number of grains per unit area mostly depended on the number of grains/spike and its components, spikelets/spike and grains/spikelet. The lack of effect of flowering time on the number of grains/spike was due to a compensation between the number of spikelets/spike and the number of grains/spikelet. This compensation was much lower at the spring-planting site (Mexico-South) because the number of spikelets/spike recorded at this site was lower than that of the other two sites. The large environmental effect on the number of spikelets/spike reported by Arjona et al. [39] was identified here to be strongly associated in all sites with the photo-thermal units from terminal spikelet to flowering.

The study of the relationship between allele combinations and yield stability showed that allele combination *Ppd-A1b/Ppd-B1a* (SI) was the only one with a regression slope [9] significantly different from 1, thus revealing that it conferred the lowest yield stability but the greatest responsiveness to good growing conditions. Allele combination GS100-*Ppd-A1a*/*Ppd-B1a* (I0I) had a regression slope higher than 1, also suggesting good yield responsiveness to environmental improvements. Although the regression slope line is the most widely used index to measure stability due to its straightforward interpretation, additional information is provided by other indices. The lowest value of the Lin and Binns [10] superiority measure (*Pi*) recorded on combination I0I suggests good performance at all experimental sites. According to Shukla’s [11] stability variance (*σ*^2^) and Wricke’s [12] ecovalence (*W_i_*^2^), the low relative values shown by allele combinations *Ppd-A1b*/*Ppd-B1b* (SS) and GS105-*Ppd-A1a*/*Ppd-B1a* (I5I) suggested the greatest yield stability in the static sense. With regard to Kang’s [13] YSi ratio, which gives the same weight to yield and stability, allele combination I0I stands out for its better yield performance, albeit being a little less stable than combinations SS and I5I. Its yield performance and responsiveness compensated for its lower stability parameters in the temperate environments of this study. The fact that the allele combination SS is among the most stable will have relevance in higher latitudes (above 45°) than those studied in this research. However, for environments below 45° latitude, which represents the majority of the durum wheat-growing area, the SS allele combination stability is generally accompanied with the lowest yield, making it unsuitable for the temperate areas. Würschum et al. [33] found significant differences in durum genotypes from different European countries attributable to different copy number variation at *Ppd-B1*, with early types corresponding to the southern countries. Unlike Würschum et al., we did not find polymorphism for *Ppd-B1* [39], but the linked marker used in our study revealed implications for yield components and adaptation features when considered in combination with *Ppd-A1*.

As climate change will increase the variability of environmental conditions, extracting the maximum yield possible for each situation will improve food security in the future [53]. Obtaining precise stability values of new varieties is expensive and requires data from many environments [54], which sometimes is not feasible at the selection step in breeding programs. Such analyses are generally obtained once a cultivar is released or right before, when extensive multi-environment data are available. Therefore, it may be useful for breeders to use any tool considered a reliable indicator of yield stability. Information regarding allelic composition at *Ppd-1*, including copy number variation as described in Würschum et al. [33], could be used to enhance the breeder’s capacity to predict yield stability. Huang et al. [55] found that genomic selection for stability of yield and heading date was possible in winter wheat, while this was not true for several quality traits. The results of the current study support the findings of Huang et al. [55] as allele combinations affecting heading and flowering date, and linked to yield stability, may be important targets for genomic selection. Moreover, molecular markers for *Ppd-A1* and *Ppd-B1* allele variants are currently available at acceptable cost to be used for marker-assisted selection (MAS) in breeding programs. While our findings could be extrapolated to other polyploid species, such as bread wheat, the diploid ones such as barley or some varieties of rye should rely on other genetic features to achieve high stability. Muhleisen et al. [54] indicated that for barley there are not suitable traits to be used as indicators of yield stability. They showed that the use of hybrids instead of inbred varieties would be advantageous to improve dynamic stability in barley. Therefore, yield stability indicators seem to be very specific.

As photoperiod-sensitive genotypes are generally grown at high latitudes, it was expected that genotypes carrying the *Ppd-A1b* allele would be well adapted to northern Spain. However, even at this site genotypes carrying allele combination I0I (GS100-*Ppd-A1a*/*Ppd-B1a*) yielded 9.1% more than those carrying combinations SS (*Ppd-A1b*/*Ppd-B1b*) or SI (*Ppd-A1b*/*Ppd-B1a*). This is consistent with the success in northern Spain of the CIMMYT-derived germplasm that is characterised by very little photoperiod sensitivity and has been bred for wide adaptation within the low latitude environments. The high values of P_i_ and Wricke’s ecovalence (*W_i_*^2^), the significance of the Shukla’s stability, *σ*^2^, and the negative values of Kang’s YS_i_ ratio observed in allele combinations SI and I5S revealed a low yield stability for them. Among the studied stability indicators, Kang’s criterion may be one of the most useful for breeders, since it has a balanced weight of dynamic and static stabilities. However, every criterion has its merits and its shortcomings, this fact being the reason for the existence of a great number of criteria to measure yield stability [7].

Allele combinations carrying alleles conferring the same response to photoperiod at both *Ppd-1* loci proved to be the most stable, with I0I (GS100-*Ppd-A1a*/*Ppd-B1a*) being the most suitable for the study sites in terms of yield and yield stability. It has been reported that allele GS100 is rare among modern durum wheat varieties and has not been found in landraces [32,56]. Its more systematic integration into breeding programs could represent an avenue for reducing flowering time while possibly increasing yield and yield stability within the low-latitude range of the studied sites.

## 4. Materials and Methods

### 4.1. Plant Material

This work was conducted with 23 spring-growth habit durum wheat (*Triticum turgidum* L. ssp. *durum*) genotypes, including two commercial cultivars (‘Simeto’, medium to late-flowering) and ‘Anton’ (late-flowering) and 21 inbred lines developed from crosses between 10 parents with divergent flowering dates. Late and early genotypes were crossed and self pollinated as bulks from F_1_ to F_3_. Lines with contrasting heading time were selected within crosses at F_4_ and advanced to reach homozygosis. The molecular characterisation for the *Vrn-1*, *Vrn-3* and *Ppd-1* loci, showed that genotypes were monomorphic for *Vrn-A1*, *Vrn-B1* and *Vrn-B3*, and *Ppd-B1* copy number variation was not detected [39]. A summary of the allele combinations for the *Ppd-A1* and *Ppd-B1* loci carried by the genotypes of the collection is presented in Table 1 and the pedigrees and molecular characterisation of each genotype are shown in Supplementary Table S2. Samples of genotypes’ seeds are available upon request.

### 4.2. Experimental Field Setup

Field experiments were performed during five years (2007, 2008, 2010, 2011 and 2012) at three irrigated sites of contrasting latitude: North-eastern Spain (Lleida, 41° 38′ N), Mexico-North (Ciudad Obregón, 27° 21′ N) and Mexico-South (El Batán Experimental Station, Texcoco, 19° 31′ N). A detailed description of the sites and their environmental characteristics is found in Villegas et al. [36] and is summarised in Supplementary Table S3. The experiments comprised plots of 12 m^2^ replications, organised as randomised complete block designs with three replications. Sowing density was fitted at each site to obtain around 200 plants/m^2^. Agronomic management was also carried out according to the usual practices at each site, avoiding pests, diseases and weeds that would interfere with the results. The 10 experiments in Spain and Mexico-North were sown from 19 November to 23 December depending on the year, while the five experiments in Mexico-South were spring-planted (from 17 to 28 May) as usual in this site. Temperature (daily maximum, minimum, and mean values) and solar radiation were obtained from meteorological stations within or near the experimental fields. Daily photoperiod including twilight was estimated according to Forsythe et al. [57]. In order to characterise the environments, the following environmental variables were calculated for each plot according to its phenology: average daily recorded mean temperature during the period from emergence to flowering (Tmean_EF_,°C) and from flowering to physiological maturity (Tmean_FM_,°C); average maximum daily temperatures from five days before to five days after the flowering date (Tmax_F_,°C); mean daily solar radiation from emergence to flowering (Rmean_EF_, MJ/m^2^ day), from double ridge to flowering (Rmean_DRF_, MJ/m^2^ day) and from flowering to physiological maturity (Rmean_FM_, MJ/m^2^ day); accumulated solar radiation from emergence to flowering (Rac_EF_, MJ/m^2^); mean day length from emergence to flowering (DLmean_EF_, h) and from flowering to physiological maturity (DLmean_FM_, h); and finally photo-thermal units from terminal spikelet to flowering (PTU_TSF_, °C h), calculated according to Dalezios et al. [58].

### 4.3. Data Recording

Growth stages (GS) at emergence (GS10), flowering (GS65) and physiological maturity (GS87, indicated by the loss of green colour in the spike peduncle) were recorded on each plot according to the Zadoks’ scale [59] during the five years of the study. Additionally, the stages of double ridge, terminal spikelet [60] and heading (GS55) were also determined in the experiments conducted in 2011, 2012 and 2013, as described in Arjona et al. [39]. A developmental stage was recorded on a plot when at least 50% of the plants had reached it. Each field plot was divided in half, with one part being used for sampling and the other half left intact for yield assessment through mechanical harvest at ripening. Grain yield (GY, g/m^2^) is expressed on a dry weight basis; GW (mg/grain) was determined from a random sample of 200 dried grains per plot; and GN, referred to here as grains/m^2^, was computed as the ratio between GY and GW. In the experiments conducted in 2010, 2011 and 2012, a sample of a 1 m-long section of a representative central row of each plot was pulled out at maturity. The spikes were counted, threshed, and their grains were also counted. In five randomly-chosen main spikes, spikelets were counted and spikelets/spike were obtained. At the Spanish site, fertile florets were also counted in the same spikes. Grains/spike were calculated by dividing the number of grains of the sample by the spike number. Grains/spikelet were computed as the ratio between grains/spike and spikelets/spike. In Spain, the grain setting was calculated as the proportion of fertile florets/spike that became grains at maturity.

### 4.4. Statistical Analysis

A principal component analysis (PCA) was conducted using JMP 13 Pro^®^ [61], and a correlation matrix was calculated with the environmental variables that were not strongly correlated among them, and with the above-mentioned yield-related traits averaged for each experiment across genotypes and replications (n = 15). Combined ANOVAs across experiments were performed using the General Linear Model (GLM) procedure of the SAS^®^ software [62], including year and genotype as random factors in the model. The sum of squares of the genotype effect was split into differences attributable to *Ppd-A1*/*Ppd-B1* allele combinations and differences between genotypes within allele combinations. The error term used to test *Ppd-1* allele combinations was the sum of squares of genotype within each allele combination. Means were compared using Fisher’s protected least significant differences (LSD) method at *P* = 0.05. To calculate the genetic variation within allele combinations (considered in this study as earliness per se in the case of phenology), the following model was considered for each experiment and run in JMP 13 Pro^®^ [61]:

Yij = ACi + G(AC)ij + Rj + Eij (1)

where:

Yij is the trait studied for genotype i in experiment j;
ACi is the effect of allele combination for genotype i, considered as a fixed effect;
G(AC)ij is the genotype effect within its allele combination for genotype i on experiment j, considered as a random effect;
Rj is the effect of the replicate on experiment j, considered as a random effect; and
Eij is the experimental error of genotype i in experiment j, considered as a random effect.

In order to analyse the earliness caused by *Eps*, their effect on flowering time was detached from that of the *Ppd-1* genes. For this purpose, the best linear unbiased predictors (BLUPs) for the G(AC)ij effect were extracted from the standard least squares/restricted maximum likelihood (REML) method at each site and year. Pearson’s correlation analysis was run on these values by site and year with PROC CORR of the SAS^®^ software [62]. Five stability indices were calculated for each allele combination. The slope (*b*) of the join regression analysis [9], and the Lin and Binns [10] superiority measure (*Pi*) were computed with SAS^®^ software [62]. Shukla’s [11] stability variance (*σ*^2^), Wricke’s [12] ecovalence (*W_i_*^2^) and Kang’s [13] non-parametric yield stability measure (YS_i_) were calculated following Dia et al. [63] using the R package “Agricolae” [64] executed in R [65].

## 5. Conclusions

The wide range of environmental conditions characteristic of the three sites where the experiments were carried out allowed strong relationships to be detected between environmental variables and yield formation traits. Large GN per unit area was associated with low temperatures and radiation before flowering. In addition, a large number of spikelets/spike resulted from high solar radiation from double ridge to heading and also from a high radiation/temperature ratio from terminal spikelet to flowering.

The range of flowering dates resulting from the different allele combinations at *Ppd A1/Ppd-B1* loci were not sufficient to generate yield variations, owing to a compensation between GN and GW. A consistent effect of allele *Ppd-B1b* on GN per unit area was observed as a result of an increase in the number of grains/spike. Allele interaction caused the intensity of this effect to depend on the allele variant present at *Ppd-A1*. Allele combination GS100-*Ppd-A1a*/*Ppd-B1a* was the most suitable for the range of environments considered here in terms of yield and yield stability. Within the variability of flowering dates observed in this study, even when caused by *Ppd-1* genes or *Eps*, a delay in flowering date had no effect on the number of grains per unit area or for its components, i.e., spikes per unit area and grains/spike. However, in all experiments, late flowering genotypes had lighter grains and in nearly all of them a delay in flowering time decreased yield.

The analysis of yield stability classified the allele combinations into two groups. Combinations carrying alleles conferring the same response at both loci, i.e., GS100-*Ppd-A1a*/*Ppd-B1a* (I0I), GS105-*Ppd-A1a*/*Ppd-B1a* (I5I) and *Ppd-A1b*/*Ppd-B1b* (SS), resulted in greater yield stability than combinations carrying alleles conferring a different photoperiod sensitivity response at both loci, i.e., GS105-*Ppd-A1a*/*Ppd-B1b* (I5S) and *Ppd-A1b*/*Ppd-B1a* (SI).

## Figures and Tables

**Table 1:** Allele combinations for *Ppd-A1* and *Ppd-B1* loci present in the durum wheat collection used in this study.

*Ppd-A1*		*Ppd-B1*		Allele Combination Acronym	Number of Genotypes
Allele ^(1)^	Photoperiod Response	Allele	Photoperiod Response		
*Ppd-A1b*	Sensitive	*Ppd-B1b*	Sensitive	SS	5
*Ppd-A1b*	Sensitive	*Ppd-B1a*	Insensitive	SI	5
*GS105-Ppd-A1a*	Insensitive	*Ppd-B1b*	Sensitive	I5S	4
*GS105-Ppd-A1a*	Insensitive	*Ppd-B1a*	Insensitive	I5I	6
*GS100-Ppd-A1a*	Insensitive	*Ppd-B1b*	Sensitive	I0S ^(2)^	1
*GS100-Ppd-A1a*	Insensitive	*Ppd-B1a*	Insensitive	I0I	3

^(1)^ Terminology following Wilhelm et al. [25]. ^(2)^ Further discarded from the study due to low genotype number.

**Table 2:** Percentage of the sum of squares of the analysis of variance (ANOVA) model for phenology and yield-related traits of 23 durum wheat genotypes grown at three sites during five years.

Source of Variation	Df	Days Emergence-Flowering	Days Flowering-Maturity	Grains/m^2^ (GN) ^(1)^	Grain Weight (GW, mg) ^(2)^	Grain Yield (GY, g/m^2^)
Site	2	86.4 ***	43.6 *	42.4 ***	14.1 *	50.5 ***
Year	4	2.8 ^ns^	7.1 ^ns^	5.0 ^ns^	5.1 ^ns^	10.5 *
Site × year	8	6.1 ***	27.0 ***	2.9 ***	8.9 ***	3.8 ***
Genotype	22	3.1 ***	4.4 *	27.5 ***	55.8 ***	11.0 ***
Between allele combinations	4	1.5 *	0.3 ^ns^	11.5 *	20.5 ^ns^	2.7 ^ns^
Within allele combinations	18	1.6 ***	4.1 ***	16.0 ***	35.3 ***	8.3 ***
Genotype × Site	44	1.0 ***	3.6 ***	2.3 ^ns^	3.4 ***	3.4 **
Between allele combinations × Site	8	0.2 ^ns^	2.2 ***		1.2 *	1.2 *
Within allele combinations × Site	36	0.8 ***	1.4 ^ns^		2.2 ***	2.2 *
Genotype × Year	88	0.2 ***	3.9 *	3.5 ^ns^	2.9 *	3.4 ^ns^
Genotype × Site × Year	176	0.3 ***	5.5 ***	6.8 ***	3.9 ***	7.4 ***
Between allele combinations × Site × Year	32	0.1 ^ns^	1.3 ^ns^	1.6 ^ns^	0.9 ^ns^	1.9 *
Within allele combinations × Site × Year	144	0.2 ***	4.2 ***	5.2 ***	3.0 ***	5.5 ***
Block	30	0.01 ***	0.5 ***	1.8 ***	0.8 ***	1.8 ***
Residual	651	0.1	4.3	7.9	5.1	8.1

^ns^: non-significant; * *P* < 0.05; ** *P* < 0.01; *** *P* < 0.001. ^(1)^ GN was calculated as GY/GW; ^(2)^ GW was determined with 200 grains.

**Table 3:** Means comparison for the spike components in a set of 23 durum wheat genotypes grouped according to five allele combinations at *Ppd-A1* and *Ppd-B1* loci and tested during five years at three sites of contrasting latitude.

Allele Combination	Grains/Spike				Spikelets/Spike				Grains/Spikelet			
	Spain	Mexico-North	Mexico-South	Mean	Spain	Mexico-North	Mexico-South	Mean	Spain	Mexico-North	Mexico-South	Mean
I0I	36.8 ^b^	36.3 ^b^	32.9 ^a^	35.4 ^b^	16.8 ^c^	17.3 ^a^	15.4 ^c^	16.5 ^c^	2.2 ^a^	2.1 ^a^	2.1 ^a^	2.1 ^ab^
I5I	36.2 ^b^	34.4 ^b^	29.9 ^a^	33.5 ^b^	17.4 ^bc^	17.4 ^a^	16.0 ^bc^	16.9 ^bc^	2.1 ^a^	2.0 ^a^	1.9 ^a^	2.0 ^b^
I5S	47.4 ^a^	47.7 ^a^	38.9 ^a^	44.6 ^a^	19.8 ^a^	20.5 ^a^	17.1 ^ab^	19.1 ^a^	2.4 ^a^	2.3 ^a^	2.3 ^a^	2.3 ^a^
SS	38.9 ^b^	40.1 ^b^	33.4 ^a^	37.4 ^b^	19.1 ^ab^	20.3 ^a^	17.7 ^a^	19.0 ^a^	2.1 ^a^	2.0 ^a^	1.9 ^a^	2.0 ^b^
SI	36.6 ^b^	40.4 ^b^	32.8 ^a^	36.6 ^b^	18.9 ^ab^	19.9 ^a^	17.3 ^ab^	18.7 ^ab^	2.0 ^a^	2.0 ^a^	1.9 ^a^	2.0 ^b^

Means within columns with different letters are significantly different for a LSD test at *P* < 0.05. See Table 1 for acronym description.

**Table 4:** Pearson correlation coefficients between phenology and yield related traits of 23 durum wheat genotypes grown in 15 environments. This analysis was conducted with the best linear unbiased predictors (BLUPs) of each genotype after removing the *Ppd-1* allele combination effect. GN: grain number, GW: grain weight, GY: grain yield.

Year	Days from Emergence to Flowering			Days from Flowering to Maturity		
	GN	GW	GY	GN	GW	GY
*Spain*						
2007	0.23 ^ns^	−0.61 **	−0.71 ***	−0.19 ^ns^	0.45 *	0.46 *
2008	0.27 ^ns^	−0.58 **	−0.57 **	−0.22 ^ns^	0.45 *	0.45 *
2010	0.33 ^ns^	−0.67 ***	−0.58 **	−0.15 ^ns^	0.31 ^ns^	0.28 ^ns^
2011	0.55 **	−0.62 **	0.01 ^ns^	0.36 ^ns^	−0.01 ^ns^	0.38 ^ns^
2012	−0.03 ^ns^	−0.50 *	−0.57 **	0.28 ^ns^	0.03 ^ns^	0.37 ^ns^
*Mexico-North*						
2007	0.16 ^ns^	−0.54 **	−0.37 ^ns^	0.16 ^ns^	−0.14 ^ns^	0.06 ^ns^
2008	0.09 ^ns^	−0.57 **	−0.70 ***	−0.14 ^ns^	0.13 ^ns^	0.13 ^ns^
2010	−0.09 ^ns^	−0.41 ^ns^	−0.55 **	−0.03 ^ns^	−0.06 ^ns^	−0.14 ^ns^
2011	−0.04 ^ns^	−0.59 **	−0.70 ***	−0.14 ^ns^	0.68 ***	0.56 **
2012	−0.34 ^ns^	−0.74 ***	−0.95 ***	0.34 ^ns^	0.57 **	0.81 ***
*Mexico-South*						
2007	−0.17 ^ns^	−0.62 **	−0.81 ***	0.24 ^ns^	0.06 ^ns^	0.36 ^ns^
2008	−0.08 ^ns^	−0.47 *	−0.76 ***	0.15 ^ns^	0.06 ^ns^	0.32 ^ns^
2010	0.74 **	−0.51 *	0.67 ***	−0.17 ^ns^	0.03 ^ns^	−0.18 ^ns^
2011	0.39 ^ns^	−0.78 ***	−0.49 *	−0.04 ^ns^	0.26 ^ns^	0.40 ^ns^
2012	0.29 ^ns^	−0.62 **	−0.23 ^ns^	0.02 ^ns^	−0.09 ^ns^	0.02 ^ns^

^ns^: non-significant; * *P* < 0.05; ** *P* < 0.01; *** *P* < 0.001.

**Table 5:** Pearson correlation coefficients between spike components and the number of days to flowering and grains/m^2^ of 23 durum wheat genotypes grown in nine environments. This analysis was conducted with the BLUPs of each genotype after removing the *Ppd-1* allele combination effect.

Year	Days from Emergence to Flowering				Grains/m^2^ (GN)				
	Spikes/m^2^	Grains/Spike	Spikelets/Spike	Grains/Spikelet	Spikes/m^2^	Grains/Spike^1^	Spikelets/Spike		Grains/Spikelet
*Spain*									
2010	0.14 ^ns^	−0.04 ^ns^	0.83 ***	−0.29 ^ns^		−0.12 ^ns^	0.59 ***	0.22 ^ns^	0.59 **
2011	0.46 *	0.10 ^ns^	0.74 ***	−0.37 ^ns^		0.38 ^ns^	0.67 ***	0.66 ***	0.21 ^ns^
2012	−0.41 ^ns^	0.26 ^ns^	0.66 ***	−0.12 ^ns^		0.32 ^ns^	0.72 ***	0.53 **	0.57 **
*Mexico-North*									
2010	0.31 ^ns^	−0.22 ^ns^	0.70 ***	−0.74 ***		0.44 *	0.58 **	0.45 *	0.28 ^ns^
2011	−0.30 ^ns^	0.21 ^ns^	0.70 ***	−0.41 *		0.36 ^ns^	0.75 ***	0.44 *	0.63 **
2012	−0.50 *	0.02 ^ns^	0.75 ***	−0.54 ***		0.32 ^ns^	0.65 ***	0.10 ^ns^	0.72 ***
*Mexico-South*									
2010	0.07 ^ns^	0.63 **	0.12 ^ns^	0.68 ***		0.15 ^ns^	0.69 ***	0.48 *	0.55 **
2011	−0.41 ^ns^	0.56 **	0.80 ***	0.34 ^ns^		0.06 ^ns^	0.83 ***	0.55 **	0.74 ***
2012	−0.14 ^ns^	0.45 *	0.41 *	0.32 ^ns^		0.54 ***	0.78 ***	0.41 *	0.74 ***

^ns^: non-significant; * *P* < 0.05; ** *P* < 0.01; *** *P* < 0.001.

**Table 6:** Stability indices calculated for grain yield of 23 durum wheat genotypes grouped according five allele combinations for *Ppd-A1* and *Ppd-B1* loci and grown at three contrasting latitudes during five years. See Table 1 for acronym description.

Allele Combination	Grain Yield (GY, g/m^2^)	Regression Slope (*b*)	Superiority Measure (*Pi*)	Shukla’s Stability (*σ*^2^)	Wricke’s Ecovalence (*W_i_*^2^)	Kang’s Yield-Stability (YS_i_)
I0I	600 ^a^	1.12 ^a^	2542 ^e^	755 ^ns^	1563	+8
SS	554 ^a^	0.96 ^b^	63966 ^d^	−365 ^ns^	219	+5
I5I	547 ^a^	0.90 ^b^	80228 ^c^	−80 ^ns^	561	+2
I5S	542 ^a^	0.87 ^b^	103535 ^b^	4143 **	5628	−7
SI	516 ^a^	1.19 ^a,(1)^	139795 ^a^	3754 *	5162	−5

^(1)^ Significantly different from 1; *: Significantly different from 0 at *P* < 0.05; **: Significantly different from 0 at *P* < 0.01; +: Stable, and −: Unstable allele combinations according to Kang’s yield stability criterion. Means within columns with different letters are significantly different for a LSD test at *P* < 0.05.

## Supplementary Materials

The following are available online at https://www.mdpi.com/2223-7747/9/12/1723/s1, Table S1: Values used to construct Figure 3: Means comparison corresponding for phenology and yield related traits in a set of 23 durum wheat genotypes grouped according to five allele combinations at *Ppd-A1* and *Ppd-B1* loci and tested during five years at three sites of contrasting northern latitude. Table S2: Pedigrees and allelic combinations for *Ppd-A1* and *Ppd-B1* loci harboured by the genotypes used in the study. Table S3: Geographic and environmental descriptors for the three experimental sites. Table S4: Mean comparison of grain setting in a set of 23 durum wheat genotypes grouped according to five allele combinations at *Ppd-A1* and *Ppd-B1* loci and tested during three years in Spain.
