# Supplementary material for: The Effect of Photoperiod Genes and Flowering Time on Yield and Yield Stability in Durum Wheat
Source: Plants (Basel). 2020 Dec 7;9(12):1723. doi: 10.3390/plants9121723 (PMC7762236; doi:10.3390/plants9121723)
Supplement: Supplementary file 1 [file plants-09-01723-s001.pdf]

plants

MDPI

Supplementary Material

Table S1. Values used to construct Figure 3: Means comparison corresponding for phenology and yield related traits in a set of 23 durum wheat genotypes grouped according to five allele combinations at *Ppd-A1* and *Ppd-B1* loci and tested during five years at three sites of contrasting northern latitude. Means within columns with different letters are significantly different for a LSD test at $p<0.05$. See Table 1 for acronym description.

Allele combination	Days emergence-flowering				Days flowering-maturity				Grains/m^2 (GN)*				Grain weight (GW, mg)**				Grain yield (GY, g/m^2)			
	Spain	Mexico-North	Mexico-South	Mean	Spain	Mexico-North	Mexico-South	Mean	Spain	Mexico-North	Mexico-South	Mean	Spain	Mexico-North	Mexico-South	Mean	Spain	Mexico-North	Mexico-South	Mean
I0I	130^{b}	83^{b}	60^{b}	92^{c}	38^{a}	43^{ab}	49^{a}	43^{a}	18148^{ab}	12588^{b}	11270^{b}	14002^{b}	43.2^{a}	50.6^{a}	42.9^{a}	45.6^{a}	744^{a}	605^{a}	455^{a}	600^{a}
I5I	132^{ab}	84^{b}	63^{ab}	93^{bc}	36^{a}	43^{a}	50^{a}	43^{a}	16396^{b}	11254^{b}	10987^{b}	12879^{b}	43.6^{a}	50.2^{a}	41.8^{a}	45.2^{a}	677^{a}	534^{a}	430^{a}	547^{a}
I5S	135^{a}	90^{ab}	66^{ab}	97^{abc}	36^{a}	41^{abc}	49^{a}	42^{a}	20706^{a}	15540^{a}	14627^{a}	16958^{a}	32.8^{b}	37.4^{b}	32.1^{b}	34.1^{a}	642^{a}	544^{a}	441^{a}	542^{a}
SS	136^{a}	95^{a}	67^{ab}	99^{ab}	36^{a}	39^{bc}	52^{a}	43^{a}	18004^{ab}	13012^{ab}	12276^{ab}	14431^{ab}	40.4^{ab}	45.2^{ab}	37.6^{ab}	41.1^{a}	682^{a}	548^{a}	433^{a}	554^{a}
SI	136^{a}	97^{a}	70^{a}	101^{a}	37^{a}	39^{c}	51^{a}	42^{a}	17359^{b}	12040^{b}	10859^{b}	13419^{b}	42^{a}	45.2^{ab}	35.9^{ab}	41.0^{a}	682^{a}	504^{a}	363^{a}	516^{a}

*: Calculated as GY/GW; **: Determined with 200 grains.

Table S2. Pedigrees and allelic combinations for *Ppd-A1* and *Ppd-B1* loci harboured by the genotypes used in the study.

Pedigree	Allele variant at *Ppd-A1*	Allele variant at *Ppd-B1*	Allelic combination acronym
2905-13.93.04//DUKEM_12/2*RASCON_21	*GS100 Ppd-A1a*	*Ppd-B1a*	I0I
MEGADUR//DUKEM_12/2*RASCON_21	*GS100 Ppd-A1a*	*Ppd-B1a*	I0I
MEGADUR//DUKEM_12/2*RASCON_21	*GS100 Ppd-A1a*	*Ppd-B1a*	I0I
2716-25.94.01/3/SNITAN	*GS105 Ppd-A1a*	*Ppd-B1a*	I5I
2716-25.94.01/3/SNITAN	*GS105 Ppd-A1a*	*Ppd-B1a*	I5I
2805-49.94.02/GUANAY	*GS105 Ppd-A1a*	*Ppd-B1a*	I5I
2905-13.93.04//CADO/BOOMER_33	*GS105 Ppd-A1a*	*Ppd-B1a*	I5I
DURABON//SOOTY_9/RASCON_37	*GS105 Ppd-A1a*	*Ppd-B1a*	I5I
DURABON//SOOTY_9/RASCON_37	*GS105 Ppd-A1a*	*Ppd-B1a*	I5I
2805-49.94.02//CADO/BOOMER_33	*GS105 Ppd-A1a*	*Ppd-B1b*	I5S
2905-13.93.04//CADO/BOOMER_33	*GS105 Ppd-A1a*	*Ppd-B1b*	I5S
2905-13.93.04//CADO/BOOMER_33	*GS105 Ppd-A1a*	*Ppd-B1b*	I5S
2905-13.93.04/SNITAN	*GS105 Ppd-A1a*	*Ppd-B1b*	I5S
2716-25.94.01/GUANAY	*Ppd-A1b*	*Ppd-B1b*	SS
2716-25.94.01/GUANAY	*Ppd-A1b*	*Ppd-B1b*	SS
2905-13.93.04//CADO/BOOMER_33	*Ppd-A1b*	*Ppd-B1b*	SS
2905-13.93.04//CADO/BOOMER_33	*Ppd-A1b*	*Ppd-B1b*	SS
2905-13.93.04/SNITAN	*Ppd-A1b*	*Ppd-B1b*	SS
2805-49.94.02//CADO/BOOMER_33	*Ppd-A1b*	*Ppd-B1a*	SI
2905-13.93.04//CADO/BOOMER_33	*Ppd-A1b*	*Ppd-B1a*	SI
2905-13.93.04//CADO/BOOMER_33	*Ppd-A1b*	*Ppd-B1a*	SI
Anton	*Ppd-A1b*	*Ppd-B1a*	SI
Simeto	*Ppd-A1b*	*Ppd-B1a*	SI

Table S3. Geographic and environmental descriptors for the three experimental sites.

Site	Spain	Mexico-North	Mexico-South
Location	Lleida	Ciudad Obregón	El Batán
Experimental station (institution)	Gimenells (IRTA)	CENEB (CIMMYT)	El Batán (CIMMYT)
Coordinates			
Latitude	41° 38'N	27° 21'N	19° 31'N
Longitude	0° 23'E	109° 54'W	98° 50'W
Altitude (m asl.)	200	40	2249
Soil characteristics			
Texture	Fine-loamy	Clay	Clay
pH	8.1	8.5	5.9
P (ppm)	16	2.4	65
K (ppm)	134	273	312
Organic matter (%)	2.4	1.2	5

Table S4. Mean comparison of grain setting in a set of 23 durum wheat genotypes grouped according to five allele combinations at *Ppd-A1* and *Ppd-B1* loci and tested during three years in Spain. Means within columns with different letters are significantly different for a LSD test at $p<0.05$. See Table 1 for acronym description.

Allele combination	Grain setting (%)	
I0I	0.62	a
I5I	0.64	a
I5S	0.63	a
SS	0.61	a
SI	0.60	a
